# Humidity control and hydrophilic glue coating applied to mounted protein crystals improves X-ray diffraction experiments

**DOI:** 10.1107/S0907444913018027

**Published:** 2013-08-17

**Authors:** Seiki Baba, Takeshi Hoshino, Len Ito, Takashi Kumasaka

**Affiliations:** a Structural Biology Group, Japan Synchrotron Radiation Research Institute (JASRI/SPring-8), 1-1-1 Kouto, Sayo, Hyogo 679-5198, Japan

**Keywords:** cryocrystallography, macromolecular crystallography, crystal mounting

## Abstract

Protein crystals are fragile, and it is sometimes difficult to find conditions suitable for handling and cryocooling the crystals before conducting X-ray diffraction experiments. To overcome this issue, a protein crystal-mounting method has been developed that involves a water-soluble polymer and controlled humid air that can adjust the moisture content of a mounted crystal. By coating crystals with polymer glue and exposing them to controlled humid air, the crystals were stable at room temperature and were cryocooled under optimized humidity. Moreover, the glue-coated crystals reproducibly showed gradual transformations of their lattice constants in response to a change in humidity; thus, using this method, a series of isomorphous crystals can be prepared. This technique is valuable when working on fragile protein crystals, including membrane proteins, and will also be useful for multi-crystal data collection.

## Abstract 2

A new crystal-mounting method has been developed that involves a combination of controlled humid air and polymer glue for crystal coating. This method is particularly useful when applied to fragile protein crystals that are known to be sensitive to subtle changes in their physicochemical environment.

## 1. Introduction

Cryopreservation of protein crystals substantially reduces radiation damage (Haas & Rossmann, 1970 ▶). However, simple cryocooling of a crystal is accompanied by possible crystal distortion caused by ice formation during the cryopreservation process owing to the presence of large solvent channels in protein crystals (Garman & Schneider, 1997 ▶). The most widely used effective technique for preventing ice formation is to replace the crystal mother liquor by an aqueous/organic mixture solution (Petsko, 1975 ▶) using a cryoloop, a simple crystal-mounting tool composed of low X-ray-absorption materials (Teng, 1990 ▶; Garman & Schneider, 1997 ▶).

Many developments have improved this technique. To improve the handling of samples or to reduce scattering noise from mounting apparatuses, a loopless mount (Kitago *et al.*, 2005 ▶; Watanabe, 2006 ▶), a crystal catcher using an adhesive (Kitatani *et al.*, 2008 ▶) and a microfabricated polyimide film (Thorne *et al.*, 2003 ▶) have been developed as alternatives to standard cryoloops. To eliminate or reduce the amount of cryoprotectants that permeate the crystals and may sometimes cause damage to them, a cryoprotectant-free procedure has been achieved by maximizing the crystal-cooling rate (Warkentin *et al.*, 2006 ▶; Pellegrini *et al.*, 2011 ▶), and a method using a high-pressure environment (Kim *et al.*, 2005 ▶) has also been reported.

Meanwhile, crystal cooling is generally thought to introduce slight changes into protein structures. Therefore, to study temperature-sensitive structures experiments at both room and cryogenic temperatures are necessary (Halle, 2004 ▶; Dunlop *et al.*, 2005 ▶; Fraser *et al.*, 2011 ▶). A capillary-free mounting system (Kiefersauer *et al.*, 2000 ▶) and a humidity-control device (Sanchez-Weatherby *et al.*, 2009 ▶) are well suited to conducting these experiments. However, these systems were originally designed to improve the resolution of protein crystals as a post-crystallization treatment, in which lattice transformation is induced by variable controlled humidity that results in the hydration or dehydration of crystals. Therefore, to maximize the effect of humid air, protein crystals should be exposed to air by removal of the mother liquor around the protein crystal. Since crystals exposed to air are fragile owing to their high sensitivity to environmental humidity changes, the applications of this technique have been limited.

To improve the stability of crystals while maintaining the applicability of capillary-free mounting, we examined crystal-coating materials and found a suitable water-soluble polymer solution: aqueous polyvinyl alcohol (PVA) glue. This substance is widely used not only as a stationery glue and as a laundry starch but also in biological applications: for instance, as a cryopreservation reagent for embryos (Nowshari & Brem, 2000 ▶) and as a mounting medium for microscopic subjects such as microbes (Omar *et al.*, 1978 ▶). Although protein crystals coated with this material showed only a small sensitivity to humidity changes, the benefits of conferring high stability and affinity to various fragile samples far outweigh this limitation. In this report, we introduce this technique, the humid air and glue-coating (HAG) crystal-mounting method, and demonstrate its success with various protein crystals.

## 2. Materials and methods

### 2.1. Water-soluble polymers

As the water-soluble polymer, we used polyvinyl alcohol (PVA) with average polymerization degrees ranging from 2000 to 4500 (PVA2000, PVA2400, PVA3500 and PVA4500). PVA2000, PVA2400 and PVA4500 were supplied by Japan VAM & POVAL Co. Ltd, Japan (product Nos. JP-20, JP-24 and JP-45, respectively). PVA3500 was purchased from Wako Pure Chemical Industries Ltd (product No. 163-16355). Aqueous solutions of PVA4500 [8%(*w*/*w*) and 12%(*w*/*w*)], PVA3500 [13%(*w*/*w*)] and PVA2400 [15%(*w*/*w*)] were dissolved in water by brief heating (363 K) and stirring.

**Figure 1:**
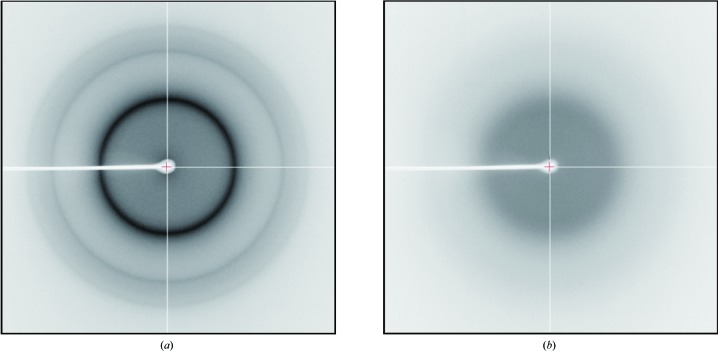
Diffraction images of flash-cooled PVA glue. (*a*) 8%(*w*/*w*) PVA4500 exposed to air of 92.3% RH. (*b*) 8%(*w*/*w*) PVA4500 exposed to air of 92.0% RH.

For flash-cooling experiments, humidity conditions that vitrify the PVA solutions themselves were examined by X-ray scattering. Initial solutions of 8%(*w*/*w*) and 13%(*w*/*w*) PVA4500 gave a powder diffraction pattern of hexagonal ice after flash-cooling without any humidity control. Similarly, glue exposed to air of 92.3% RH (relative humidity) produced a powder diffraction pattern of cubic ice (Fig. 1 ▶ *a*), but at 92.0% RH no ice ring was observed (Fig. 1 ▶ *b*). Since the final resultant concentrations of PVA are affected by vapour pressure, *i.e.* atmospheric humidity (Hwang *et al.*, 1998 ▶), it is essential to control the humidity in order to prevent the glue solution from forming ice. In contrast, the degree of polymerization (PVA2000, PVA2400, PVA3300 and PVA3500) did not seem to affect the vitrification and glues adjusted to 92% RH or below did not produce any ice rings after flash-cooling. Thus, 8%(*w*/*w*) PVA4500 was used as the mounting glue in all of the experiments if not otherwise specified.

### 2.2. The humidity-control apparatus

**Figure 2:**
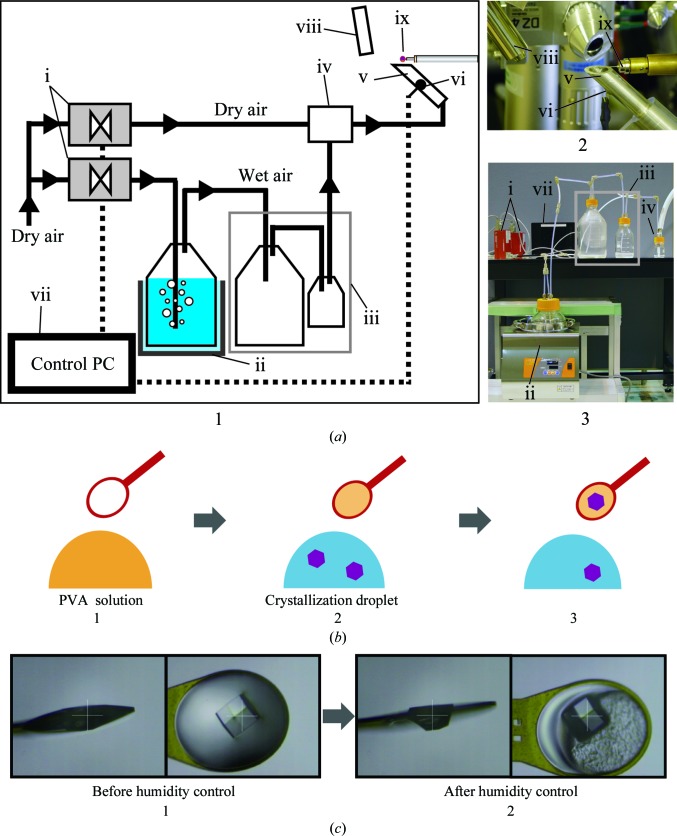
Overview of the HAG method. (*a*) The humidity-control apparatus: 1, design of the apparatus; 2, the humid air nozzle and the sample; 3, the configuration of the two-flow humidity generator. The apparatus was assembled from the following components: i, digital mass-flow controller; ii, temperature-controlled air-bubbling bottle (2 l); iii, air-cooling bottles (2 l and 500 ml); iv, air-mixing bottle (100 ml); v, humid air nozzle; vi, temperature and humidity sensor; vii, PC to control and monitor humidity through i and ii; viii, cryostream nozzle; ix, crystal sample. (*b*) Crystal-handling procedures: 1, applying the PVA glue to a cryoloop; 2, picking up a crystal; 3, steeping the crystal to be coated by the glue. (*c*) A mounted crystal: 1, just before the crystal was placed on the apparatus; 2, after exposure to air of 73.9% RH.

We developed a simple humidity-control apparatus equipped with a two-flow humidity generator (Fig. 2 ▶ *a*). Dry air was supplied by an N_2_-gas generator (Iwatani Co. Ltd, Japan) and an HOHMI YC-4R air compressor (Yaezaki Kuatsu Co. Ltd, Japan). Saturated-humidity air was produced by bubbling the dry air in a water bath kept at 301 K and cooling to room temperature (296 ± 1 K with a temperature stability of ±0.3 K h^−1^) during transportation. Next, it was mixed with the dry air and the total flow rate was brought to 3 l min^−1^ or greater using two SEC-N series digital mass-flow controllers (Horiba Ltd, Japan) controlled by a PC running Windows 7. Although this handmade humidity generator was used in this study, we confirmed that a modified version of the commercial humidity generator HUM-1 (Rigaku Co., Japan), which can produce humid air at a high flow rate, also worked for this purpose. The humidity-control gas was transported to a humid air nozzle, which was produced from a flexible Teflon tube with an inner diameter of 10 mm. The outlet temperature and the relative humidity were measured inside the humid air nozzle using an SHT71 humidity sensor (Sensirion AG, Switzerland). This humidity-control device can control the humidity range between 0.1 and 94.5% RH. Since the humidity was regulated by the mixture of gases, humidity change took place at a rate of between 0.2 and 1% RH min^−1^.

### 2.3. Experimental protocols of the HAG method

#### 2.3.1. Apparatus setup

The humidity-control apparatus was installed on the SPring-8 BL38B1 beamline equipped with a one-axis rotation goniometer (Kohzu Seiki Co. Ltd, Japan), ADSC Quantum 210 or 315 CCD detectors and a GN2 nitrogen cryostream system (Rigaku Co.). All of the following operations were performed at room temperature (296 ± 18 K with a temperature accuracy of ±0.3 K h^−1^). The humid air nozzle of the apparatus was placed under the sample (Fig. 2 ▶ *a*) and the humidity was adjusted; typically, the initial humidity was 83% RH. The cryostream nozzle was removed from the sample position to avoid interference with the humid air (Fig. 2 ▶ *a*). In the following steps performed at room temperature, the intensity of X-ray radiation should be minimized in order to reduce radiation damage. In the case of our beamline, SPring-8 BL38B1, an aluminium plate with a thickness of 600–1000 µm was used to attenuate the X-ray beam with the full photon flux of 1.17 × 10^11^ photons s^−1^ to 1/10 to 1/40 at a wavelength of 1 Å, respectively.

#### 2.3.2. Glue preparation

An aqueous glue solution consisting of 8%(*w*/*w*) PVA4500 was used except for certain crystallization conditions. PVA interacts with ions, especially multivalent anions such as tartrate, phosphate and sulfate ions, and forms a physical hydrogel. Therefore, it is often difficult to thoroughly coat the crystals owing to a varying degree of hydrogel elasticity that depends on the concentration and the valences of the ions. It is known that plasticizing reagents such as glycerol, ethylene glycol and polyethylene glycol 400 prevent gel formation and that 5%(*v*/*v*) glycerol is sufficient to reduce gel formation (Sakellariou *et al.*, 1993 ▶). Thus, we also examined three modified methods of sample handling for ionic solutions. (i) The protein crystal was soaked in reservoir solution supplemented with 5%(*v*/*v*) glycerol before transferring the crystal into the glue. (ii) Glycerol was added to the crystallization solutions to a final concentration of 5%(*v*/*v*). (iii) In the case of crystallization solutions containing a high concentration of multivalent ions (*e.g.* greater than 1 *M*), the glue was prepared with equal volumes of pure glycerol and the aqueous PVA solution.

#### 2.3.3. Crystal coating and mounting

A small amount of the glue solution was applied to and spread over a crystal-mounting loop (*e.g.* LithoLoops; Protein Wave Co.) with a typical diameter larger than the crystal (labelled 1 in Fig. 2 ▶ *b*). A crystal was directly picked up from a crystallization droplet by the glue-coated loop without removal of the mother liquor surrounding the crystal (labelled 2 in Fig. 2 ▶ *b*). The scooped crystal was kept steady for a few seconds so that it could be thoroughly coated and covered by the glue on the loop (labelled 3 in Fig. 2 ▶ *b*). Next, the loop was mounted on the diffractometer and exposed to the humid air. This operation should be completed within 10–20 s to minimize transpiration of the crystalline moisture.

#### 2.3.4. Searching for a suitable humidity condition

**Figure 3:**
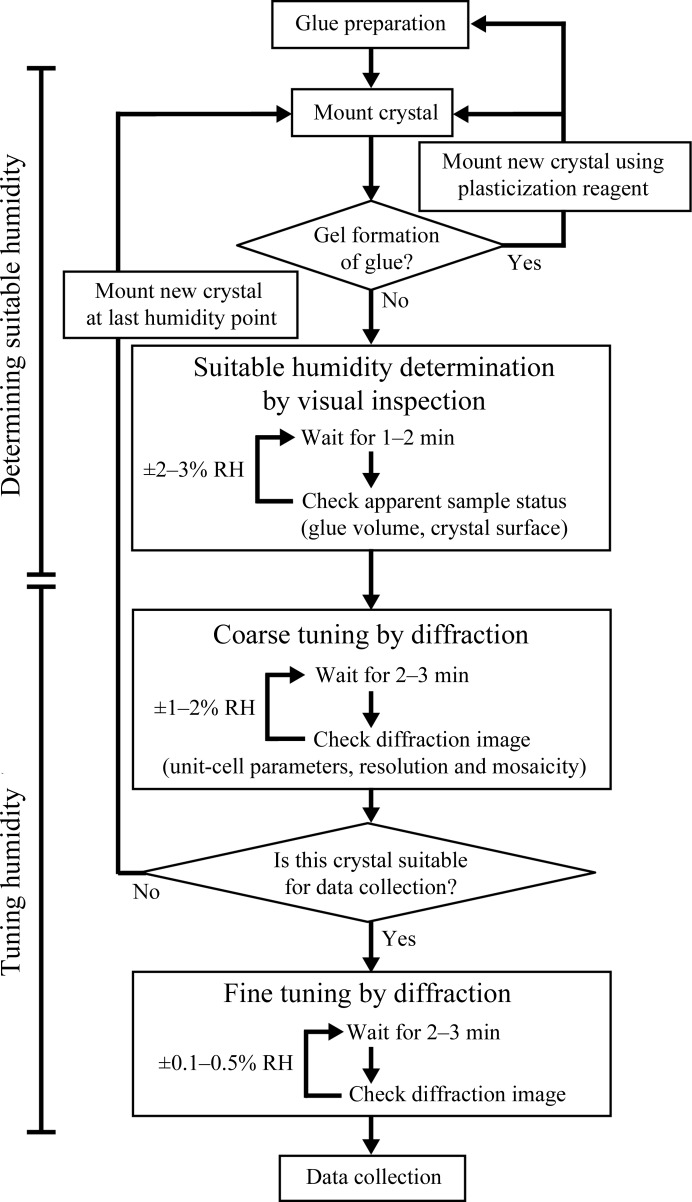
A flowchart summarizing the humidity-optimization strategy in the HAG mounting method.

A destructive hydration shock could be induced if the glue were diluted by absorbing a large amount of the droplet solution in the crystal pickup (Fig. 2 ▶ *c*). To avoid this, a suitable humidity level needs to be established immediately. The swelling of the glue and consequent formation of crystalline ice at cryogenic temperature is another concern. The protocol for coping with these problems is described in Fig. 3 ▶.

To search for a suitable humidity condition, the crystal quality was not only judged by visual inspection but was also evaluated by obtaining diffraction images, which were processed using the *HKL*-2000 suite (Otwinowski & Minor, 1997 ▶).

#### 2.3.5. Determining the suitable humidity

The suitable humidity and the extent of the safe humidity range vary from one protein crystal to another. In this step, we roughly determined the appropriate humidity range in which the crystal did not suffer from any unrecoverable damage such as fragmentation and dissolution. In most cases, these types of damage were apparent from careful observation; signs of chaps or cracks on the crystal surface and volume expansion of the glue will lead to damage to the crystal. In some cases, we also observed that the crystal surface melted in a hypotonic condition.

We started the experiments with 83.0% RH humid air and changed it in steps of ±2–3% RH. When a crystal suffered from unrecoverable damage in this step, the next crystal was mounted with a new humidity condition selected from the best apparent status from the previous experiments, as judged by analytical and algorithmic determination of the optimal value for a unimodal function against all measured data points.

#### 2.3.6. Tuning the humidity

Optimization of the suitable humidity was conducted by checking the crystal quality by obtaining one diffraction image at each scanned humidity point. Initially, a coarse optimization with steps of 1–2% RH was performed and was followed by a finer humidity optimization with steps of 0.1–0.5% RH. To ensure sufficient time to equilibrate water distribution in the whole system, the crystal diffraction check was delayed for a few minutes after each humidity change. If any irreversible damage, *i.e.* a sudden loss of resolution, increasing mosaicity or splitting of spots, was detected in the diffraction pattern, the crystal was replaced by a new crystal and the experiment was continued from the last humidity point.

Once the humidity had been optimized, it was kept constant without any further change. Before proceeding to the next step, the crystal was maintained at the optimum humidity for 15 min to complete water equilibration. Since lower humidity was preferred in many crystals, the glue was dehydrated and shrunk at optimum humidity (labelled 2 in Fig. 2 ▶ *c*).

#### 2.3.7. Flash-cooling

In order to perform cryogenic experiments at 100 K, the humid air stream was replaced by the Rigaku GN2 cryostream. Firstly, the shutter of the cryostream was closed and its nozzle was returned to the sample position. After the humid air nozzle had been removed, the cryostream shutter was immediately opened. Although the humidity range for successful cryocooling and the optimum humidity depended on the sample (Table 1 ▶), we empirically determined that the initial humidity for unknown samples should be 83.0% RH. However, the number and the variety of experiments conducted in the present study may not be extensive enough to establish a standardized condition that can apply to a wide range of different crystals.

### 2.4. Protein crystals

The following five types of crystals were prepared to validate the HAG method.

#### 2.4.1. Lysozyme

Hen egg-white lysozyme was obtained from Seikagaku Co. (product No. 100940). Lysozyme crystals were grown using hanging-drop vapour diffusion at 293 K. Hanging drops were prepared by mixing 5 µl of 35 mg ml^−1^ protein solution in 50 m*M* sodium acetate buffer pH 4.5 with an equal volume of a solution consisting of 1.6 *M* sodium chloride, 50 m*M* sodium acetate buffer pH 4.5. The volume of reservoir solution consisting of 1.0 *M* sodium chloride, 50 m*M* sodium acetate buffer pH 4.5 was 500 µl per well. For the series of experiments with the lysozyme crystals, we carefully selected crystals by size from the same batch.

#### 2.4.2. Hydrolase RsbQ

The bacterial hydrolase RsbQ from *Bacillus subtilis* was expressed and purified as described previously (Kaneko *et al.*, 2005 ▶). The new form of RsbQ crystals was grown using hanging-drop vapour diffusion at 293 K as follows: hanging drops were prepared by mixing 1 µl of 12 mg ml^−1^ protein solution in 50 m*M* HEPES buffer pH 7.5 with an equal volume of a reservoir solution consisting of 200 m*M* NaI, 20% PEG 3350, 1 m*M* reduced glutathione, 50 m*M* HEPES buffer pH 7.5. The volume of reservoir solution was 150 µl per well. Thin plate crystals initially appeared with a thickness of a few micrometres and the microseeding technique improved the thickness to approximately 100 µm. We tried cryoprotectant solutions prepared using glycerol, ethylene glycol, polyethylene glycol 400, 2-­methyl-2,4-pentane­diol and trehalose at a concentration of 25%(*w*/*v*), which was the lowest concentration to vitrify the mother liquor. However, the crystals were mechanically very fragile and sensitive to environmental changes. Indeed, the crystals gradually delaminated and crumbled once the cover slides of the hanging drops were opened.

#### 2.4.3. Insulin

Bovine insulin was obtained from Sigma–Aldrich Co. (product No. I550) and crystallized based on the method reported by Gursky *et al.* (1992 ▶). The crystals of insulin were grown using hanging-drop vapour diffusion at 293 K. Hanging drops were prepared by mixing 2 µl of 20 mg ml^−1^ protein solution in 20 m*M* sodium phosphate buffer pH 12.0 with an equal volume of a reservoir solution consisting of 1 m*M* EDTA, 0.4 *M* sodium phosphate buffer pH 10.4. The volume of reservoir solution was 500 µl per well. To avoid PVA gel formation by the phosphate ion, the crystals were soaked in reservoir solution containing 5%(*v*/*v*) glycerol.

#### 2.4.4. *Bv*RC

The bacterial membrane protein *Blastochloris viridis* photoreaction centre (*Bv*RC) crystal was provided by Dr Takayuki Odahara. The crystals of *Bv*RC were grown using sitting-drop vapour diffusion at 288 K with a small glass vessel for protein solution installed in a sealed plastic container. Sitting drops were prepared using 400 µl of 18 mg ml^−1^ *Bv*RC solution in 2.5%(*w*/*v*) triethylamine phosphate, 2.5%(*w*/*v*) 1,2,3-hexanetriol, 0.3% lauryl dimethylamine oxide (LDAO), 1.51 *M* ammonium sulfate, 60 m*M* sodium phosphate buffer pH 7.0. The volume of reservoir solution consisting of 5%(*w*/*v*) triethylamine phosphate, 1.89 *M* ammonium sulfate, 100 m*M* sodium phosphate buffer pH 7.0 was 12 ml in each setup. The glue for the HAG mount was a mixture prepared from equal volumes of pure glycerol and 8%(*w*/*w*) PVA4500 solution.

#### 2.4.5. LTC_4_S

The human membrane protein leukotriene C_4_ synthase (LTC_4_S) was provided by Dr Hideo Ago (Ago *et al.*, 2007 ▶; Saino *et al.*, 2011 ▶). The crystals of LTC_4_S were grown using sitting-drop vapour diffusion at 293 K in a 96-well crystallization plate. Drops were prepared by mixing 5–10 µl of 6 mg ml^−1^ LTC_4_S solution in 20 m*M* MES–NaOH pH 6.5, 5 m*M* glutathione, 0.04%(*w*/*v*) *n*-dodecyl-β-d-maltoside (DDM) with an equal volume of reservoir solution consisting of 0.04%(*w*/*v*) DDM, 5 m*M* glutathione, 1.6–1.7 *M* ammonium sulfate, 0.5–0.8 *M* magnesium chloride, 20 m*M* MES–NaOH buffer pH 6.5. The crystals of LTC_4_S were transferred overnight into harvesting solution consisting of 2.4 *M* ammonium sulfate, 50 m*M* glutathione, 20 m*M* MES–NaOH buffer pH 6.5 (Saino *et al.*, 2011 ▶). The glue used was the same as that used for *Bv*RC.

## 3. Results

### 3.1. The HAG method works with different types of crystals, including those of two membrane proteins

**Figure 4:**
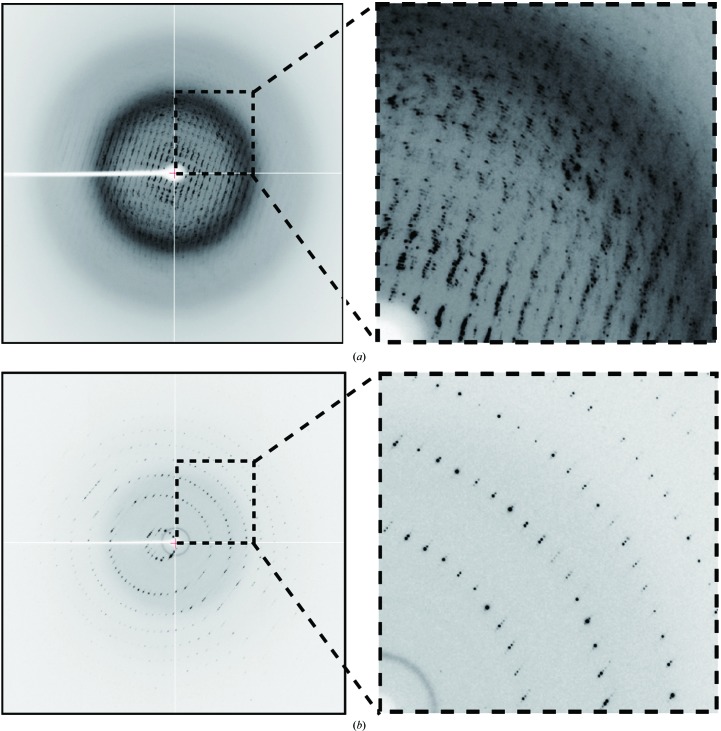
Diffraction images of bacterial hydrolase RsbQ crystals. (*a*) A diffraction image from a crystal treated using a typical cryoprotection procedure. The crystal was mounted with a cryosolution composed of the reservoir solution supplemented with 25%(*v*/*v*) glycerol and then flash-cooled. (*b*) A diffraction image from a crystal mounted with the HAG method. The crystal was mounted using 13%(*w*/*w*) PVA3500 glue with a humidity of 69.1% RH and then flash-cooled. Both samples were kept at 100 K.

We tested this method on several protein crystal samples following the procedure shown in Fig. 3 ▶ and described in §[Sec sec2]2. Using this method, most of the samples successfully underwent humidity-optimization experiments at room temperature followed by full data collection at 100 K (Table 1 ▶). The most notable result was obtained with the crystals of bacterial hydrolase RsbQ from *B. subtilis*. The crystals were mechanically fragile and were damaged by all of the soaking experiments, in which split spots were observed in diffraction images after flash-cooling (Fig. 4 ▶ *a*). By employing an HAG mount using 13%(*w*/*w*) PVA3500 glue without any other cryoprotectants and an optimized humidity of 69.1% RH, cryoprotection of the RsbQ crystals was successfully achieved and a full diffraction data set was collected (Fig. 4 ▶ *b* and Table 1 ▶). In a previous study with another crystal form, this enzyme showed some structural changes induced by the binding of propylene glycol as a cryoprotectant to the active site (Kaneko *et al.*, 2005 ▶). Therefore, it seems reasonable that the new crystal form was sensitive to these permeable cryoprotectants.

However, the HAG method initially failed when we tested crystals containing high concentrations of multivalent ions. It is known that the PVA glue immediately reacts with the ions to form a hydrogel. The stickiness of the gel made it difficult to thoroughly coat the crystals with the polymer glue, as the crystals stuck to the gel surface. To address this issue, we added glycerol, a plasticization reagent for PVA, which reduced gel formation (Sakellariou *et al.*, 1993 ▶). The insulin crystals usually required a glycerol concentration of 25%(*v*/*v*) or higher as a permeable cryoprotectant in the conventional cryocooling method, whereas they could be mounted and cryocooled with only 5%(*v*/*v*) glycerol in the HAG method. In this case the crystal was first soaked in reservoir solution supplemented with 5%(*v*/*v*) glycerol to replace the mother liquor and was then used in HAG mounting. The addition of only 5%(*v*/*v*) glycerol was sufficient to prevent gel formation by the 0.4 *M* phosphate ion contained in the mother liquor. By contrast, for both *Bv*RC and LTC_4_S crystals the addition of 5%(*v*/*v*) glycerol was insufficient to prevent gel formation because both of the crystallization conditions contained high concentrations of sulfate ions (1.9–2.5 *M*). However, addition of glycerol to the polymer glue was effective for both crystals. The glue prepared by mixing equal volumes of glycerol and 8%(*w*/*w*) PVA4500, *i.e.* a final concentration of 50%(*v*/*v*) glycerol and 4%(*w*/*w*) PVA, was successfully subjected to cryocooling without ice formation (Table 1 ▶). In the conventional cryocooling method, a *Bv*RC crystal soaked in cryoprotectant containing 30%(*v*/*v*) glycerol was damaged in only 2 min (Baxter *et al.*, 2005 ▶). However, the PVA glue and glycerol mixture preserved the crystals for over 20 min during a diffraction check at room temperature when mounted using the HAG method.

### 3.2. Humidity change causes reversible lattice transformation of lysozyme crystals at room temperature

**Figure 5:**
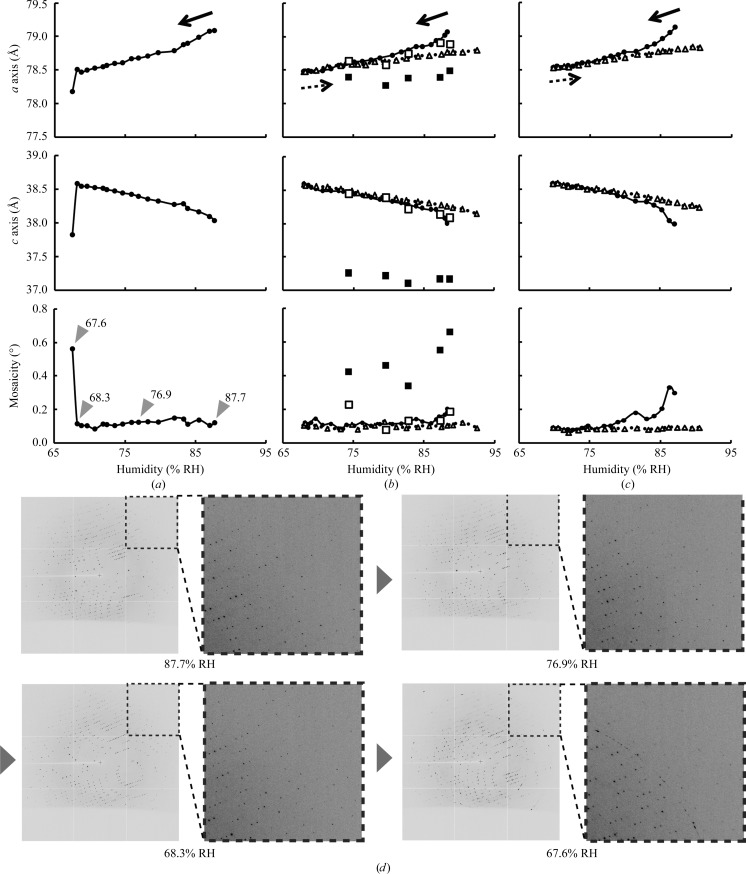
Reversible crystal lattice transformation of glue-coated lysozyme crystals subjected to humidity changes. (*a*–*c*) The effects observed on the crystallographic *a* axis (top), *c* axis (middle) and mosaicity (bottom) caused by humidity changes. (*a*) The response to a reduction in humidity. The filled circles (solid line) indicate continuous measurements of a crystal treated with humid air starting at 87.7% RH and gradually decreasing to 67.6% RH. (*b*, *c*) The response to two-way changes in humidity. The lattice-parameter changes during the humidity-reduction process are plotted as filled circles. (*b*) Humidity reduction from 88.3 to 67.9% RH. (*c*) Humidity reduction from 87.1 to 69.8% RH. In (*b*) and (*c*) the triangles (dotted line) indicate the trace of lattice-parameter changes during the humidity-recovery process up to 92.5% RH (*b*) and 90.5% RH (*c*) after the previous humidity reduction. In addition, the lattice constants observed after flash-cooling are overlaid on (*b*). For flash-cooling, five new crystals were prepared at five different humidities (74.3, 79.5, 82.7, 87.3 and 88.6% RH). Parameters obtained from the experiments conducted at room temperature and 100 K are plotted as open and filled squares, respectively. (*d*) Diffraction images collected at the humidity conditions indicated by the four wedges shown in (*a*).

In addition to the cryoprotection effects, we also investigated the transformation of lysozyme crystal lattices in response to humidity changes. Firstly, we estimated the humidity ranges in which glue-coated crystals remain stable. Tetragonal crystals of hen egg-white lysozyme were mounted by the HAG method without any additional cryoprotectants and the humidity was reduced from 87.7% RH to the catastrophic point at which the crystal was damaged (Figs. 5 ▶ *a* and 5 ▶ *d*). During the dehydration process to 68.3% RH (Fig. 5 ▶ *a*), a 0.73% shrinkage of the crystallographic *a* axis and a 1.45% stretching of the *c* axis were simultaneously observed. With further dehydration from 68.3 to 67.6% RH the crystal suddenly cracked and the mosaicity surged (Figs. 5 ▶ *a* and 5 ▶ *d*). Rehydration with increased humidity did not lead to recovery of the crystal from cracking.

**Figure 6:**
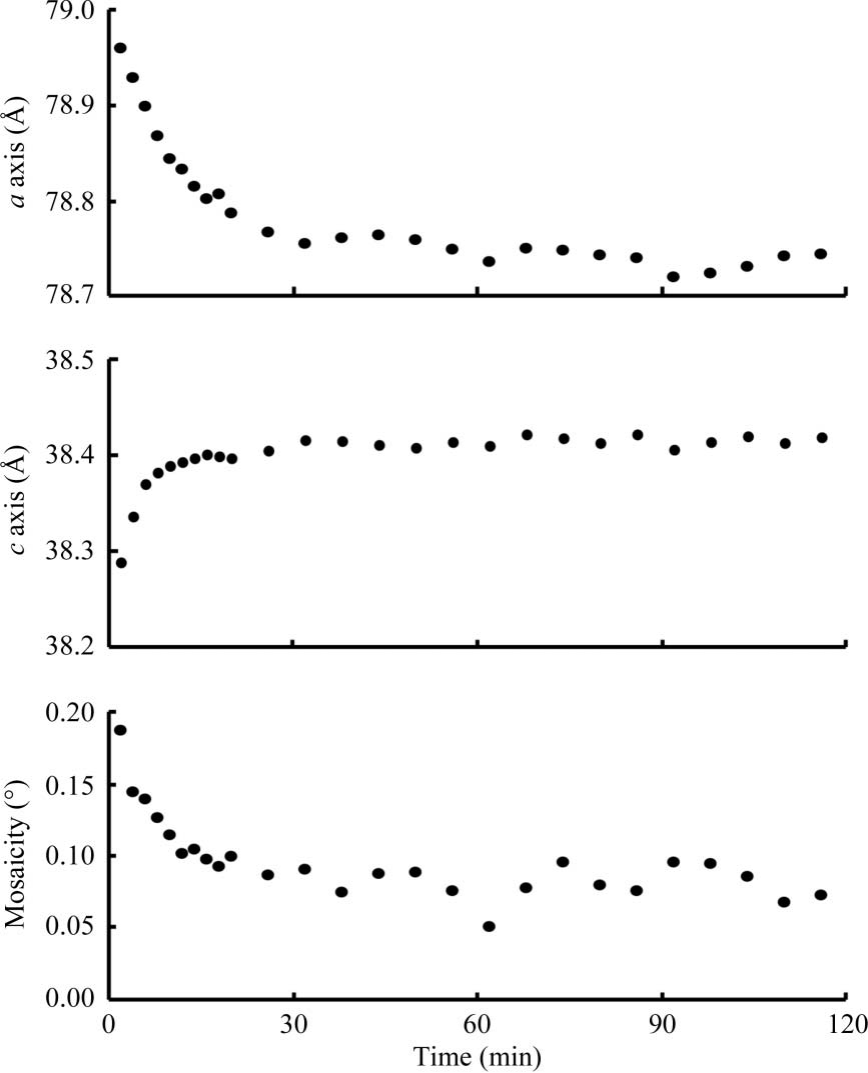
Time course of the lattice transformation of a crystal subjected to a constant humidity of 80.4% RH plotted for the crystallographic *a* axis (top), *c* axis (middle) and mosaicity (bottom).

**Figure 7:**
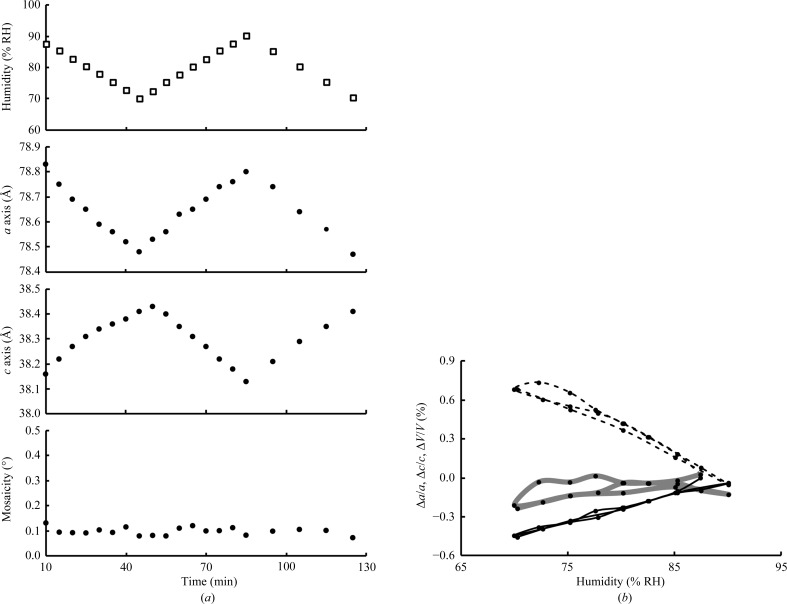
Long-term stability accompanied by reproducible lattice-constant transitions of glue-coated lysozyme crystals treated with consecutive dehydration, rehydration and re-dehydration processes. (*a*) Time course of the transformation of a crystal subjected to the following programmed humidity changes. In the first stage of the program, the relative humidity was kept at 87.4% RH for 10 min after the lysozyme crystal had been mounted. Next, dehydration (87.4 to 70.0% RH) and subsequent hydration (70.0 to 90.1% RH) experiments were conducted with a stepwise humidity change at a rate of 2.5% RH per 5 min. Finally, re-dehydration (90.1 to 70.3% RH) was applied with a humidity-change rate of 5% RH per 10 min. (*b*) The reversible changes in the lattice constants in response to humidity changes. The relative changes of the crystallographic *a* axis (Δ*a*/*a*) and *c* axis (Δ*c*/*c*) and of the unit-cell volume (Δ*V*/*V*) are plotted with solid, dotted and grey bold lines, respectively.

The reversibility of the response to dehydration was estimated in the next two experiments, in which the humidity was reduced from 88.3 to 68.1% RH immediately before the catastrophic point and was returned to 92.5% RH (Figs. 5 ▶ *b* and 5 ▶ *c*). The dehydration process gradually led to lattice transformation along the *a* and *c* axes to 78.51 and 38.35 Å, respectively. Moreover, during the following rehydration process the lattice parameters inversely traced the transitions that were observed in the dehydration process. This showed a linear relationship between the humidity and the lattice constants, although a small nonlinear response and increased mosaicity were observed in the first dehydration steps. Such a nonlinear response was also observed by exposing a crystal to constant humidity (Fig. 6 ▶). It typically took 20–30 min for the moisture to reach equilibrium between the humid air and the mounted sample, which initially contained excess moisture derived from the glue. However, once the excess moisture had been removed the crystal responded uniformly to a change in humidity and was safely maintained over 2 h in the experiment that involved successive dehydration, rehydration and re-dehydration (Fig. 7 ▶).

### 3.3. Humidity affects the diffraction data quality of flash-cooled crystals

The effect of the HAG mount for cryocooling was examined using five lysozyme crystals, each with a different humidity condition, in which no ice-ring patterns were observed but for which the mosaicities increased after cryocooling (Table 2 ▶ and Fig. 5 ▶ *b*). In particular, the samples exposed to high humidity (87.3–88.6% RH) showed higher mosaicity (0.55–0.66°) than the others (<0.46°). In terms of the resolution, the data set with the highest resolution was obtained from the 82.7% RH condition, which is in the middle of the tested humidity range (Table 2 ▶). Both extremes in humidity may possibly induce hypertonic or hypotonic stress of the crystals. As well as desiccation, we also observed that a lysozyme crystal was cracked by exposure to a high humidity of around 90% RH for 5 min or longer. In addition, there was an alternative limitation in the cryogenic experiments; *i.e.* a solution of only PVA treated with humidity above 92% RH gave ice-ring diffraction on cryocooling. It has been reported that the water vapour pressure of aqueous PVA (molecular mass 88 kDa) at concentrations of about 60% or below remains approximately at the saturated pressure of water vapour at 304.15 K (Hwang *et al.*, 1998 ▶). Thus, the PVA concentration might reach about 60%(*w*/*v*) by exposure to unsaturated humid air, leading to successful vitrification.

## 4. Discussion

Our results demonstrate the high feasibility of the HAG method for crystal mounting and cryocooling. We speculate upon several possible reasons for the feasibility and efficacy of this method.

(i) The nature of retaining moisture and buffering moisture exchange. The glue prevents direct exchange of water vapour between the crystal and the atmosphere. Instead, the glue releases its own moisture, rather than that of the crystal, to the dry atmosphere during crystal handling or humidity changes. This moderate moisture-exchange system protects fragile crystals from a hydration or dehydration shock. In addition, the hypotonic property of the polymer solution retains the moisture of a crystal. The osmotic pressure of the glue solution depends on the concentration of its solute and is also affected by the polymerization degree of the solute polymer according to the Flory–Huggins theory; a longer polymer gives a lower osmotic pressure at a given vapour pressure. Indeed, the osmolality of the glue is almost negligible (<10 mmol kg^−1^) compared with that of the crystallization solution of lysozyme (osmolality >2000 mmol kg^−1^).
(ii) High viscosity and slow diffusion. The high viscosity of the glue guards the crystals from mechanical stress and slows the diffusion of solvents, solutes and PVA itself. The high viscosity may retard changes in the chemical environment surrounding a crystal. The effects might also mitigate osmotic shocks (Sugiyama *et al.*, 2012 ▶).
(iii) Affinity for crystallization solutes. The glue might provide a similar chemical condition to the crystallization drop by absorbing the crystallization solution during crystal pickup. Even in dried conditions, the absorbed chemicals might be trapped by the glue through interaction with the hydroxyl groups of PVA, preventing the condensation of chemicals inside the crystal.
(iv) Suppression of ice crystals. Similarly to permeable cryoprotectants, the hydrophilic polymer suppresses the formation of ice crystals in the solution around the crystals. Moreover, the hydrophilic glue can absorb the excess crystallization solvent that usually contributes to ice formation. In this sense, the use of the polymer glue is different from oil coating, in which case a small amount of the excess solvent may be left at the crystal–oil border.

Through the humidity-control experiments, the glue proved to be suitable for fixing protein crystals on a cryoloop (Fig. 2 ▶ *c*). However, glue-coated lysozyme crystals showed a different response to that of the uncoated crystals to humidity changes (Dobrianov *et al.*, 2001 ▶; Salunke *et al.*, 1985 ▶; Kodandapani *et al.*, 1990 ▶; Kiefersauer *et al.*, 2000 ▶), in which a large change in lattice constants (maximally 10%) and hysteretic responses were observed. Instead, our results are similar to the observation reported in soaking experiments with a higher concentration of sodium chloride (López-Jaramillo *et al.*, 2002 ▶); that is, the response was small (less than a 1% change in lattice constants), the *c* axis expanded after dehydration and a hysteretic response was not observed. The response to humidity changes was reproducible regardless of the crystal orientation, which is different from the experiments with the uncoated crystals. These responses of the crystals in the HAG method were rather subtle and the mosaicity was almost constant. Therefore, this process might be regarded as an elastic deformation induced by a smaller stress derived from osmotic pressure.

However, a mosaicity change was often observed in the initial steps of the mounting procedure (Figs. 5 ▶ and 6 ▶). Because a relatively high mosaicity was reduced by dehydration in these cases, some part of this change might be caused by the hypotonic effect of the glue on the crystals owing to excess moisture in the glue or inappropriate initial humidity. Therefore, this undesirable effect should be further mitigated by adjustment of the osmolality of the glue and optimization of the initial humidity.

Meanwhile, the mechanism that causes the catastrophic damage upon dehydration remains unclear (Fig. 5 ▶ *a*). In the hypertonic condition, the polymer concentration exponentially correlates with the osmotic pressure. In addition to the pressure, mechanical force induced by dehydration of the PVA hydrogel and salt displacement might cause significant stress to crystals. In some cases, the glue surface became lumpy and salt crystals appeared during the dehydration processes. The surface roughness of the glue might reflect the formation of PVA hydrogel, in which physical networks of polymer molecules are formed by hydrogen bonds and ionic inter­actions together with polar or ionic solutes. To avoid unre­coverable damage, it might be necessary to optimize the humidity and the amount of the plasticizing reagents.

The controllability of the osmotic pressure will aid crystallographers to manage lattice constants and isomorphism when dealing with a series of crystals at room temperature. This feature might be useful for multi-crystal data collection using synchrotrons or X-ray free-electron lasers (Kern *et al.*, 2012 ▶). Since the flash-cooling process is not completely under our control, the HAG method does not assure isomorphism in cryocooled crystals but only provides fixation of initial conditions for cryocooling. Thus, hierarchical cluster analysis will be helpful for this purpose (Giordano *et al.*, 2012 ▶). Moreover, the hydrophilic glue will not work for preventing ice formation in solvent channels of protein crystals, similar to immiscible oils used for cryoprotection (Kwong & Liu, 1999 ▶). Recently, multicomponent mixtures of cryoprotectants have successfully provided extended stabilization during cryoprotection to allow longer soaking periods without crystal cracking or dissolving (Vera & Stura, 2013 ▶). Thus, combinatory use of multicomponent permeable cryoprotectants such as polyols and cryosalts would increase the flexibility of the method.

## 5. Conclusions

The new crystal-mounting method, the HAG method, is an effective way of handling fragile protein crystals and is compatible with experiments at both room and cryogenic temperatures. Furthermore, treatment of samples with proper humidity could produce desired lattice constants, potentially resulting in improved isomorphism for multi-crystal data collection using a series of crystals. This feature is required for experiments with radiation-sensitive crystals or when conducting experiments at room temperature using a synchrotron or an X-ray free-electron laser X-ray source. We are in the process of further improvement of the method.

## Figures and Tables

**Table 1:** Experimental conditions and diffraction data statistics for all of the examined crystals All X-ray diffraction data were collected at 100 K. Values in parentheses are for the highest resolution shell.

	Hydrolase RsbQ	Insulin	*Bv*RC	LTC_4_S
Glue composition	13%(*w*/*w*) PVA3500	8%(*w*/*w*) PVA4500	4%(*w*/*w*) PVA4500 and 50%(*v*/*v*) glycerol	4%(*w*/*w*) PVA4500 and 50%(*v*/*v*) glycerol
Optimized humidity (% RH)	69.1	82.9	83.2	83.4
Space group	*P*2_1_2_1_2_1_	*I*2_1_3	*P*4_3_2_1_2	*F*23
Unit-cell parameters (Å)	*a* = 42.30, *b* = 43.05, *c* = 256.98	*a* = 77.74	*a* = 220.58, *c* = 113.26	*a* = 167.52
Mosaicity (°)	0.33	0.44	0.33	0.30
Resolution (Å)	50.00–1.40 (1.42–1.40)	50.00–1.53 (1.56–1.53)	50.00–1.85 (1.92–1.85)	50.00–2.70 (2.80–2.70)
Multiplicity	14.0 (14.3)	21.8 (21.7)	10.1 (7.8)	11.3 (7.5)
Completeness (%)	96.8 (73.9)	99.9 (100.0)	99.8 (99.4)	100.0 (100.0)
*R*_merge_ † (%)	5.8 (42.8)	4.5 (38.7)	6.1 (49.7)	13.1 (49.5)
Average *I*/σ(*I*)	52.5 (4.0)	96.0 (12.9)	58.4 (4.3)	53.3 (5.5)

† *R*_merge_ = 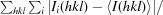 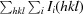, where *I_i_*(*hkl*) is the observed intensity of the *i*th measurement of reflection *hkl* and 〈*I*(*hkl*)〉 is the mean intensity of reflection *hkl* calculated after scaling.

**Table 2:** Experimental conditions and diffraction data statistics of lysozyme crystals Values in parentheses are for the highest resolution shell. The maximum resolution of each data set was determined as that where the CC_1/2_ (Karplus & Diederichs, 2012 ▶) was around 0.8 in the highest resolution shell. The values of CC_1/2_ were calculated with the *PHENIX* package (Adams *et al.*, 2010 ▶).

Optimized humidity (% RH)	74.3	79.5	82.7	87.3	88.6
Unit-cell parameters (Å)					
*a*	78.39	78.27	78.38	78.39	78.49
*c*	37.26	37.22	37.10	37.17	37.17
Mosaicity before/after cryocooling (°)	0.23/0.43	0.081/0.46	0.14/0.34	0.12/0.55	0.19/0.66
Resolution (Å)	50.00–1.18 (1.20–1.18)	50.00–1.19 (1.21–1.19)	50.00–1.13 (1.15–1.13)	50.00–1.15 (1.17–1.15)	50.00–1.15 (1.17–1.15)
Multiplicity	6.3 (3.5)	6.6 (6.5)	6.9 (6.8)	6.6 (6.5)	6.8 (6.6)
Completeness (%)	97.0(75.9)	99.7 (100.0)	99.9 (99.8)	99.9 (99.8)	98.7 (99.6)
*R*_merge_ † (%)	4.6 (49.5)	4.5 (67.1)	4.6 (65.9)	5.1 (65.8)	6.0 (81.5)
Average *I*/σ(*I*)	24.1 (2.8)	19.5 (2.6)	20.9 (2.7)	19.3 (2.6)	18.0 (2.3)
CC_1/2_	0.999 (0.814)	0.999 (0.806)	0.999 (0.796)	0.999 (0.810)	0.999 (0.807)

† *R*_merge_ = 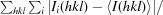 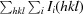, where *I_i_*(*hkl*) is the observed intensity of the *i*th measurement of reflection *hkl* and 〈*I*(*hkl*)〉 is the mean intensity of reflection *hkl* calculated after scaling.
